# The potential of strigolactones to shift competitive dynamics among two *Rhizophagus irregularis* strains

**DOI:** 10.3389/fmicb.2024.1470469

**Published:** 2024-10-18

**Authors:** Malin Klein, Corentin Bisot, Loreto Oyarte Gálvez, Vasilis Kokkoris, Thomas S. Shimizu, Lemeng Dong, James T. Weedon, Harro Bouwmeester, E. Toby Kiers

**Affiliations:** ^1^Section of Ecology and Evolution, Amsterdam Institute for Life and Environment, Vrije Universiteit Amsterdam, Amsterdam, Netherlands; ^2^Plant Hormone Biology, Swammerdam Institute for Life Sciences, University of Amsterdam, Amsterdam, Netherlands; ^3^Physics of Behavior, AMOLF Institute, Amsterdam, Netherlands; ^4^Section of Systems Ecology, Amsterdam Institute for Life and Environment, Vrije Universiteit Amsterdam, Amsterdam, Netherlands

**Keywords:** strigolactones, arbuscular mycorrhizal fungi, rhizosphere, intraspecific variation, intraspecific interaction, germination, GR24, 5-deoxystrigol

## Abstract

Strigolactones are phytohormones that influence arbuscular mycorrhizal fungal (AMF) spore germination, pre-symbiotic hyphal branching, and metabolic rates. Historically, strigolactone effects have been tested on single AMF strains. An open question is whether intraspecific variation in strigolactone effects and intraspecific interactions can influence AMF competition. Using the *Rhizophagus irregularis* strains A5 and C2, we tested for intraspecific variation in the response of germination and pre-symbiotic growth (i.e., hyphal length and branching) to the strigolactones GR24 and 5-deoxystrigol. We also tested if interactions between these strains modified their germination rates and pre-symbiotic growth. Spore germination rates were consistently high (> 90%) for C2 spores, regardless of treatment and the presence of the other strain. For A5 spores, germination was increased by strigolactone presence from approximately 30 to 70% but reduced when grown in mixed culture. When growing together, branching increased for both strains compared to monocultures. In mixed cultures, strigolactones increased the branching for both strains but led to an increase in hyphal length only for the strain A5. These strain-specific responses suggest that strigolactones may have the potential to shift competitive dynamics among AMF species with direct implications for the establishment of the AMF community.

## 1 Introduction

Arbuscular mycorrhizal fungi (AMF) are a class of symbiotic soil fungi that associate with over 70% of land plants (Brundrett and Tedersoo, 2018). These fungi forage the soil for nutrients, mainly inorganic phosphorus, and trade resources to the plants in exchange for photosynthetically derived carbon in the form of sugars and lipids (Keymer and Gutjahr, 2018). As obligate biotrophs, the life cycle of AMF is influenced by their hosts at various stages, such as germination, hyphal growth and colonization. While a host can be colonized by many AMF species simultaneously, past work has shown that AMF species and strains compete for access to host roots and have evolved specific life history traits to increase their colonization success (Hart and Reader, 2002a; Serghi et al., 2021).

One important factor in determining colonization success is the order of arrival. By arriving first to a newly growing root, AMF species can facilitate subsequent colonization of a host by additional (related) individuals, and even reduce the abundance of competing strains or species (Werner and Toby Kiers, 2015). Accordingly, the dynamics of root colonization are determined by factors influencing the timing and extent of AMF spore germination and hyphal elongation and branching in response to rhizosphere signals from the plant. Rhizosphere signals are specific compounds that convey information about the sender to the recipient, with the aim to elicit a change in the recipient’s behavior (van’t Padje et al., 2016).

Strigolactones are a diverse class of plant signals that have important dual functions. They act as phytohormones in within-plant signaling and also act as signaling molecules to initiate symbiosis with beneficial microbes in the rhizosphere (Al-Babili and Bouwmeester, 2015). Under phosphate starvation, the biosynthesis and exudation of strigolactones into the soil are induced, which is especially important for plant survival under this stress (Yoneyama et al., 2007a; Yoneyama et al., 2007b; López-Ráez et al., 2008). Studies have also shown that strigolactone exudation profiles can significantly differ between plant species and even between genotypes (Yoneyama et al., 2008; Mohemed et al., 2018; Li et al., 2023). This variation has been shown to affect the recruitment of bacteria into the root microbiome (Aliche et al., 2020; Kim et al., 2022). Additionally, different strigolactones display a large difference in the hyphal branching inducing activity in AMF (van der Heijden et al., 1998; Akiyama et al., 2010). This suggests a potential role as a selective force that could promote colonization by AMF with more desirable symbiotic function (Klironomos, 2000).

In the past decades, the effects of strigolactones, including synthetic analogues, have been well-studied on a variety of AMF at different stages of their life cycle. The synthetic strigolactone analogue GR24 is known to increase spore germination of *Rhizophagus irregularis*, *R. clarus,* and *Claroideoglomus claroideum* (Besserer et al., 2006), but no significant germination response was found in *Gigaspora margarita* and *G. rosea* (Besserer et al., 2006; Kountche et al., 2018; Tanaka et al., 2022). Both *Gigaspora* species, however, show increased hyphal branching shortly after exposure to GR24, 5-deoxystrigol, and/or root exudates of legumes containing a strigolactone blend (Akiyama et al., 2005; Gomez-Roldan et al., 2008; Yoneyama et al., 2008). Similarly, *R. irregularis* showed increased hyphal branching in germinating spores in response to GR24, while the overall germ tube length was decreased (Taulera et al., 2020). Other known, but less well-studied intracellular effects, are increased mitochondrial density, mitosis, cell proliferation, and respiration in *G. rosea* and *R. irregularis* in response to GR24 and root exudates (Besserer et al., 2006, 2008). In plant mutants that are impaired in strigolactone biosynthesis, AMF colonization is decreased or absent for both *G. rosea* (Gomez-Roldan et al., 2007, p. 200) and *R. irregularis* (Gomez-Roldan et al., 2008; Gutjahr et al., 2012; Taulera et al., 2020). For some of these plant mutants, this phenotype can be partially recovered by external GR24 application. Root colonization of wild type plants, however, cannot be increased by external strigolactone application as reported for *G. margarita*, *R. irregularis*, and *Funneliformis mosseae* (Yoshida et al., 2012; Kountche et al., 2018).

These insights into AMF-strigolactone dynamics are derived from studies using a single species and strain. It is known that AMF species vary considerably in their ecological strategies and life history traits (Hart et al., 2001; Hart and Reader, 2002b), which can differ even within a species (Serghi et al., 2021). The model species *R. irregularis,* for example, has homokaryotic and dikaryotic strains, meaning that they harbor one or two nucleotypes, respectively (Ropars et al., 2016; Kokkoris et al., 2021). Strains of these two categories show general differences in germination, foraging behavior, and colonization abilities (Serghi et al., 2021). To understand how plant signaling affects AMF colonization, it is therefore crucial to know to what extent strigolactone responses differ between AMF strains and how strains potentially affect one another. This is an important area of research as the rhizosphere is a highly competitive environment (Chepsergon and Moleleki, 2023) and the release of strigolactones by plants has the potential to change the competitive dynamics of AMF. It is likely that AMF may respond differently to strigolactones in the presence of other competitors. In particular, the germination of AMF spores may be either favored or reduced depending on the competitive environment in which the strigolactones are released. No research has yet investigated intraspecific (i.e., inter-strain) interactions of AMF in the pre-symbiotic germination process. We hypothesize that AMF show strain-specific responses to certain strigolactones, and that these responses are influenced by the presence of potential competitors.

To test these hypotheses, we used a semi-automated high-resolution imaging platform to closely track spores and emerging hyphae over 2 weeks. We used this approach to investigate the effects of the strigolactones 5-deoxystrigol and GR24 on the germination response of spores of two *R. irregularis* strains (dikaryotic A5 and homokaryotic C2) both individually as well as in mixed culture. We also examined the effect of both strigolactones on the AMF pre-symbiotic life history traits (i.e., total hyphal length and branches per germinated spore). We asked the following questions: (1) How does intraspecific variation affect strigolactone-mediated germination response and pre-symbiotic growth in *R. irregularis*? (2) How do intraspecific interactions affect spore germination and pre-symbiotic growth in the presence of strigolactones?

## 2 Materials and methods

### 2.1 Fungal material

To test the effects of intraspecific competition, we used *R. irregularis* dikaryotic strain A5 (DAOM 664344, MAT3 and MAT6) and homokaryotic strain C2 (DAOM 664346, MAT6). We chose these strains due to their distinct life history strategies based on their genetic organization (homokaryotic vs. heterokaryotic) (Serghi et al., 2021) We also wanted strains that share a similar mating type (MAT) locus to ensure a level of genetic similarity. With this selection we did not aim to identify differences between the two genetic groups, a task that would require the use of multiple homokaryotic and dikaryotic strains, but instead wanted to capture some of the large functional intraspecific variation existing within *R. irregularis*. The spores of A5 and C2 were originally obtained from *in vitro* lab cultures from the Sanders Lab (UNIL, Switzerland) and have been grown for 5+ years on Ri T-DNA transformed carrot roots (*Daucus carota*). To separate spores from media, we dissolved the media in sterile 10 mM sodium citrate at room temperature (Doner and Bécard, 1991), and then manually separated the spores by pulling apart clusters using sterile needles and a stereomicroscope in a laminar flow. We washed the spores 3x with sterile water to remove remaining media residues and stored them at 4°C until use.

### 2.2 Strigolactones

The synthetic strigolactone analogue GR24 is widely used in AMF and symbiosis studies (see references above). The natural strigolactone 5-deoxystrigol is produced by a wide range of plants and has been repeatedly shown to be of high importance for AMF colonization (Akiyama et al., 2005; Yoneyama et al., 2007a; Yoneyama et al., 2008; Akiyama et al., 2010; Ueno et al., 2018). Rac-GR24 was purchased from Biosynth (Bratislava, Slovakia) (+)-5-deoxystrigol was kindly provided by Dr. Floková, University of Amsterdam. GR24 and 5-deoxystrigol stocks were dissolved in acetone (10^−3^ M) and added to the autoclaved medium to a final concentration of 10^−7^ M (Besserer et al., 2006; Kountche et al., 2018; Tanaka et al., 2022). For the control plates, we incorporated the same volume of acetone into the autoclaved medium.

### 2.3 Experimental treatments

We designed an *in vitro* assay to expose spores of A5 and C2 to 5-deoxystrigol and GR24. To document both strains’ responses individually as well as their potential interaction, we included single-strain and mixed-strain plates. We prepared medium of 0.4% (w/v) Phytagel supplemented with 3 mM MgSO_4_ to facilitate the gelling (Akiyama et al., 2005; Yoneyama et al., 2008). Post autoclaving, we incorporated either GR24, or 5-deoxystrigol to a final concentration of 10^−7^ M (Besserer et al., 2006; Kountche et al., 2018, 20; Tanaka et al., 2022), or the same volume of acetone. We filled small petri plates (6 cm diameter) with 10 mL medium and added sterile circular sheets (5.5 cm diameter) of porous cellophane (Hoefer™ TE73) on top. The use of porous cellophane is an established method to ensure hyphal growth in a two-dimensional plane (Hitchcock et al., 1996; Ritz et al., 1996; Olsson et al., 2014).

**Figure 1:**
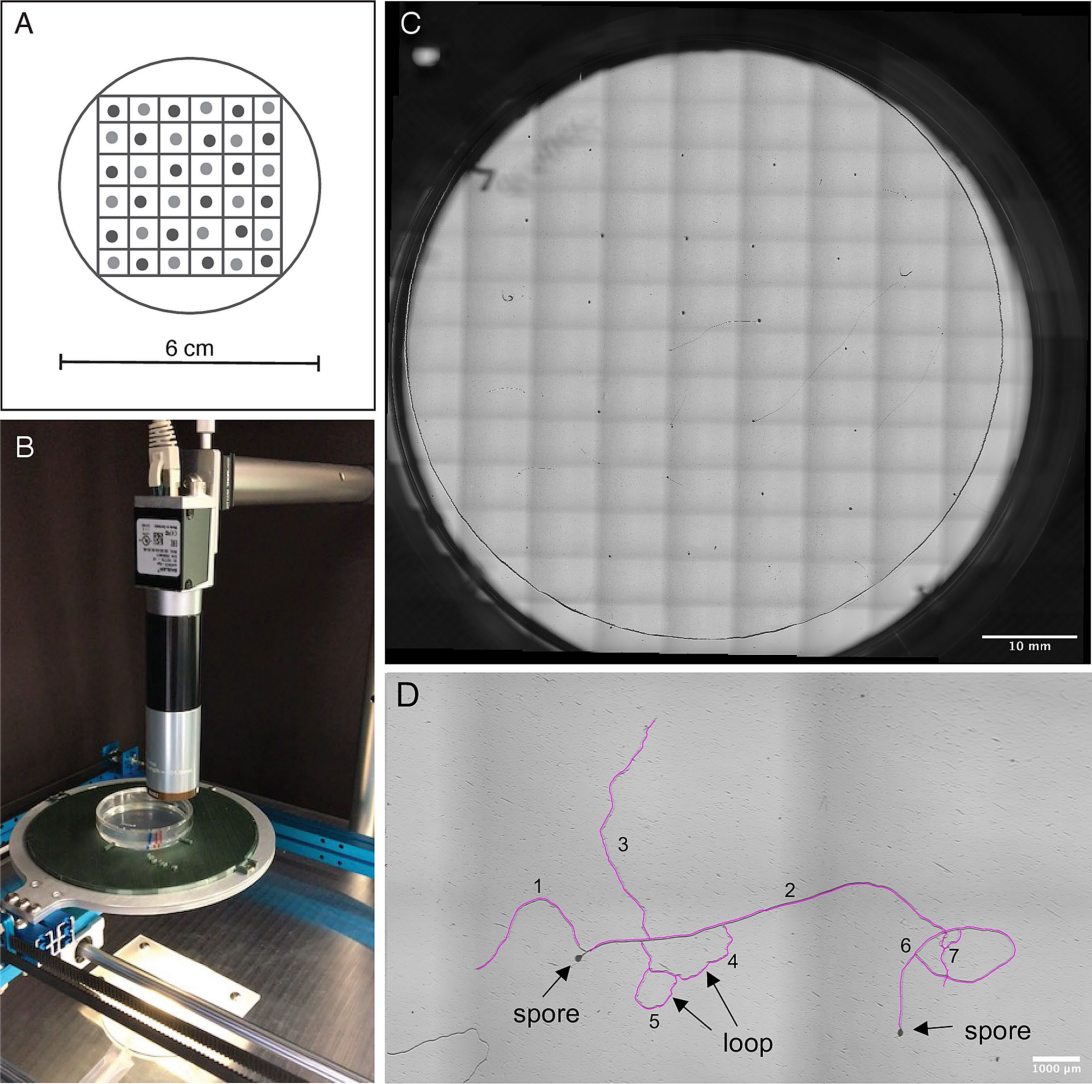
(A) Schematic figure of spore arrangement in a 6 × 6 grid on the mixed-strain plates: Spores of the two strains (shown in light and dark grey) are alternated on the grid. (B) The imaging platform consists of two motorized linear stages, a scientific camera, a 2x microscope objective lens, and a custom-built plate holder. (C) Whole plate overview. The images are taken and stitched in a 14 × 10 grid arrangement. (D) Zoom in on a plate with example of how measurements were carried out: We determined hyphal length by tracing each hypha using the NeuronJ tracer tool (tracing shown in pink). Each hypha originating from a spore (e.g., hypha 1 and hypha 2), or originating from another hypha at a Y-junction (e.g., hypha 3) was counted as a new hypha. All hyphae together contributed to the hyphal length. Hyphae that formed a loop with another hypha (hyphae 4 and 5) or with themselves (hypha 6) were also counted as individual hyphae.

We pipetted the spores individually (*ca.* 10 μL per spore) onto the cellophane using sterile equipment and a stereomicroscope in a laminar flow. In the single-strain plates we arranged the spores in a 6 × 6 grid in the center of the plate to ensure similar distances between neighbouring spores (Figure 1A). In the mixed-strain plates we kept the 6 × 6 grid with similar spacing but alternated different strains. Our aim was to place 36 or 2× 18 individual spores on each single-strain or mixed-strain plate, but due to the challenge of separating clustered spores, this varied between 31 and 39 spores for A5, and 33 and 48 spores for C2 on single-strain plates. On mixed-strain plates it varied between 16 and 21 spores for A5 and 14 to 21 spores for C2. Each strain combination (A5 single-strain, C2 single-strain, and A5-C2 mixed-strain) was exposed to the three treatment conditions (control, 5-deoxystrigol, GR24) in 6 replicate plates each. The plates were sealed with parafilm and incubated horizontally at 25°C in the dark. The experiment was carried out once with six replicate plates for each strain, treatment, and mixture combination.

### 2.4 Image acquisition and data extraction

We developed a custom-built platform to semi-automatically image each plate (Figure 1B). A full plate image consists of a grid arrangement of 140 high-resolution images (Figure 1C). Two motorized linear stages (makeblock XY-plotter robot) are used to scan the plate in x-y axis, creating a 10 × 14 position matrix. We used a scientific camera of 12MP (acA4024-8gc) together with a 2x microscope objective lens (2X *Cf* Objective, Edmund Optics). Every image has a resolution of 1.725 μm per pixel and a ~ 5 × 7 mm^2^ size. The final image is constructed using the Image Stitching plugin of ImageJ (Preibisch et al., 2009).

We imaged each plate on day 1, 2, 4, 7, 11, and 14 of spore culturing focusing on the layer of cellophane as germ tubes continue growing at this level after emergence. We verified the total number of spores per strain, with each plate representing a single biological replicate. The number of germinated spores was recorded at each imaging time point and germination rates calculated per plate by dividing number of germinated spores by total number of spores (Figure 1D). We then determined total hyphal length present by using the “add trace” tool of the NeuronJ plugin (Meijering et al., 2004) for FIJI-ImageJ v. 2.1.0/1.53c (Schindelin et al., 2012) (Figure 1D). From these measurements, we determined average hyphal length per germinated spore. As a measure for branching frequency, we counted branches (germ tube and branches of any order) on each monoculture and mix plate at 7 days post germination using the Count tool of FIJI – ImageJ v. 2.1.0/1.53c (Schindelin et al., 2012) and standardized by number of germinated spores. Since the strains germinated at different time points, the selected analysis time point of 7 days post germination was equivalent to day 11 and day 14 of the experiment for C2 and A5, respectively. Due to hyphae occasionally looping back on themselves, fusing back into the hypha they branched off from, it was impossible to distinguish between branches of different orders. We considered each hypha originating from a spore, or branching out of another hypha at a Y-junction a new hypha (Figure 1D). For example, a spore with a single emerging germ tube with one hypha branching off, was scored as having two hyphae. *R. irregularis* is known to sometimes produce several germ tubes per spore (Kokkoris et al., 2019). To identify and exclude subtending hyphae from length measurements and hyphal counts, images of days 11 and 14 were visually compared to the starting images on day 1.

### 2.5 Statistical analyses

Germination rate: to examine whether germination is mediated by strain identity (A5, C2) and/or treatment (control, 5-deoxystrigol, GR24), and/or mixed vs. monoculture conditions, we used a series of binomial generalized linear models. First, focussing on the treatment and strain effects, we modeled the effects of strain identity, treatment, and their interaction on germination success (scored per plate as proportion of germinated spores), using monoculture plates only (6 per strain × treatment combination). Secondly, we investigated the effects of growing in mixed culture. We fit binomial GLM models, with treatment, mixture (monoculture or mixed culture), and their interaction, as fixed factors, to the germination responses of each focal strain (A5 or C2) separately. This was necessary since a full mixed effects GLMM model that incorporated strain, treatment and partner identity failed to converge. For both models, comparisons between treatment combinations were performed using Tukey adjustments, and observations were weighted by the number of spores per plate, to account for some variation in number of spores per plate (see above), and the fact that mixed plates had a smaller number of spores per focal strain.

Hyphal traits: we examined whether total hyphal length and branching intensity are mediated by strain identity (A5 or C2) and/or treatment (control, 5-deoxystrigol, GR24) and/or the mixture (monoculture or mixed culture). To account for differences in development due to variation in germination time of the strains, we used the observations corresponding to 7 days post first germination per strain. We used separate linear mixed-effects models for each trait, with strain identity, treatment, mixture, and their interactions as the explanatory variables and plate ID as random factor. We also focused on each focal strain and fitted individual ANOVA models for each trait, with treatment, mixture, and their interaction as the explanatory factors. For all models, pairwise comparisons between groups were performed using Tukey adjustments. All statistical analyses were performed using R statistical software (v4.2.2) (R Core Team, 2022) with the package emmeans (version 1.8.4–1) for (planned) contrasts (Lenth et al., 2023).

## 3 Results

### 3.1 Effect of GR24 and 5-deoxystrigol on germination rate

**Figure 2:**
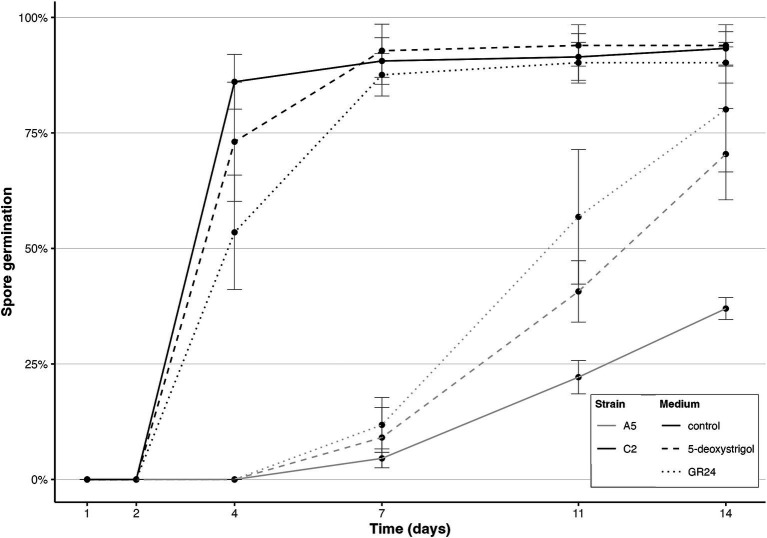
Spore germination of *R. irregularis* A5 (light grey) and *R. irregularis* C2 (dark grey) in single-strain plates over time in the control (solid line), 5-deoxystrigol (dashed line), and GR24 treatments (dotted line), *n* = 6 for each group. Error bars show the standard deviation.

We first investigated the effect of intraspecific variation on spore germination response to strigolactones. We observed that germination of the dikaryon strain A5 was slower compared to the homokaryon strain C2 (Figure 2). C2 germination started at 2–4 days and reached a high germination rate of over 87% after 7 days (mean germination proportion 90.6 ± 5% (standard deviation), 92.8 ± 5.7%, 87.6 ± 4.6% for control, 5-deoxystrigol and GR24, respectively). C2 germination further increased to over 90% at day 14 regardless of the treatment (93.3 ± 3.6%, 93.9 ± 4.5%, 90.2 ± 4.4% for control, 5-deoxystrigol and GR24, respectively). A5 first germinated between day 4 and 7 and displayed differences in germination rate in response to GR24 and 5-deoxystrigol: at day 14, germination rate in the control had reached 37 ± 2.4%, while it was 70.4 ± 9.9% and 80.1 ± 13.5% for 5-deoxystrigol and GR24, respectively.

**Figure 3:**
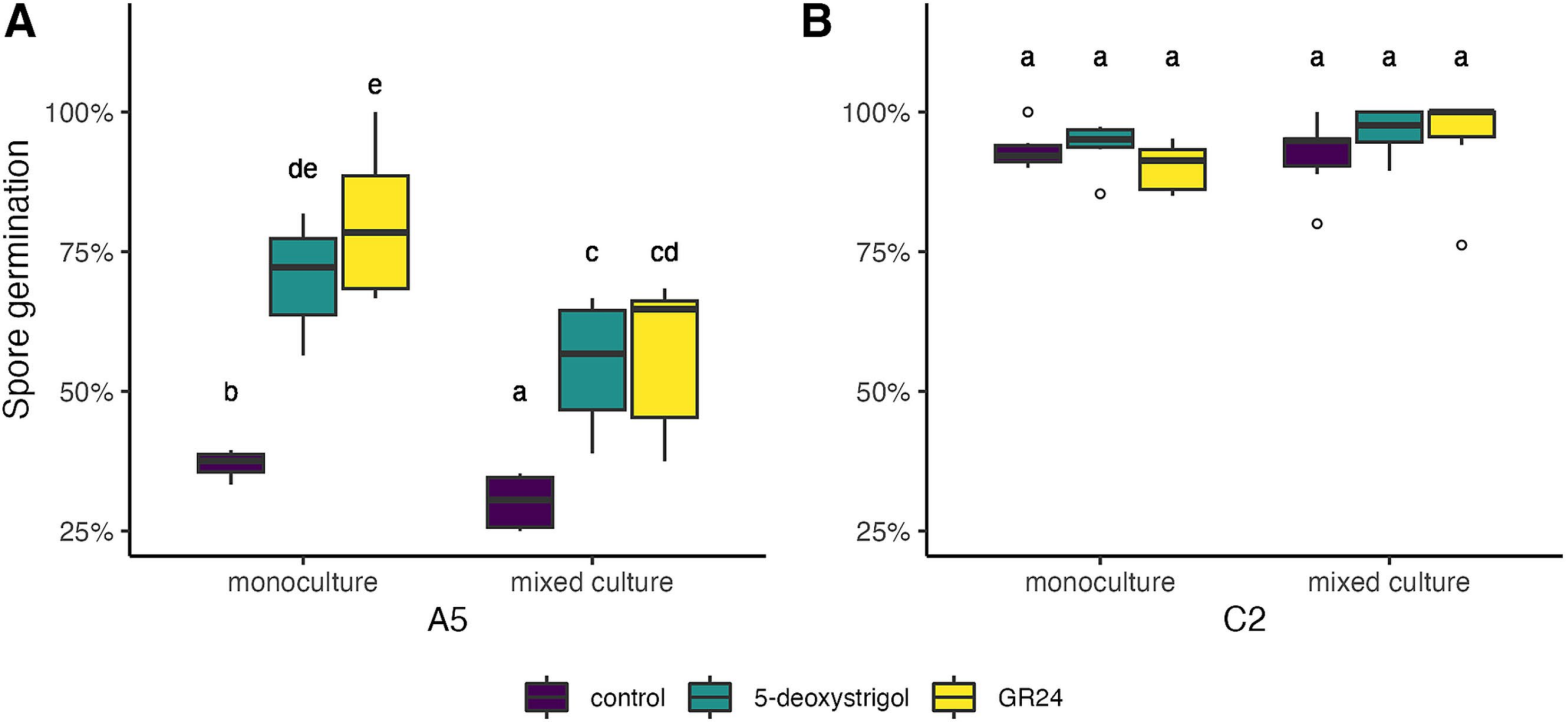
Germination of *R. irregularis* A5 (A) and C2 (B) at day 14 after start of spore culturing in response to 5-deoxystrigol (green), GR24 (yellow), and in the control (purple), in mixed or monoculture conditions. Different letters indicate pairwise statistical differences (*p* < 0.05, Tukey post-hoc test, *n* = 6 for all groups). The boxplots (*n* = 6 for each group) show the first and third quartile (boxplot edges), median (dark line), and data range (whiskers). Open circles show data outliers.

Next, we investigated whether strain and treatment affected the germination rate after 14 days of culturing (Figure 3 and Table 1). The germination in the monoculture plates after 14 days of culturing was significantly affected by strain identity (binomial GLM with likelihood ratio test, 𝜒^2^ = 194.405, 𝑑𝑓 = 1, *p* < 0.0001) and treatment (𝜒^2^ = 59.694, 𝑑𝑓 = 2, *p* < 0.0001), but also by the interaction of both (𝜒^2^ = 33.302, 𝑑𝑓 = 2, *p* < 0.0001). We compared the germination probability of the strains within treatments. The lowest germination rates were observed in A5 spores under the control treatment (37% germination, 95% Confidence Interval: 30–44%). This rate was significantly lower than all other strain × treatment combinations (Tukey test, *p* < 0.05). A5 spores subject to either of the strigolactone treatments had higher germination than controls (5-deoxystrigol: 70% [64–76%], GR24: 80% [74–85%]), with no significant differences between them. Lastly, all C2 spores had high germinations rates which were not significantly affected by treatment (*p* > 0.05, control: 93% [89–96%], 5-deoxystrigol: 94% [90–96%], GR24: 90% [86–93%]), but were higher than the rates for A5 spores in all treatments (*p* < 0.05).

**Table 1:** Germination of spores (as mean % of spores per plate +/− standard deviation) after 14 days of spore culturing for each combination of focal strain, strigolactone treatment, and mixed vs. monoculture germination conditions.

Strain	Mixture	Treatment	Spore germination (%)	across strains (monoculture)	within strain
A5	Monoculture	Control	37 ± 2.4	a	b
		5-deoxystrigol	70.4 ± 9.9	b	de
		GR24	80.1 ± 13.5	b	e
	Mixed culture	Control	27.9 ± 9.2		a
		5-deoxystrigol	54.9 ± 11.5		c
		GR24	56.8 ± 14.5		cd
C2	Monoculture	Control	93.3 ± 3.6	c	a
		5-deoxystrigol	93.9 ± 4.5	c	a
		GR24	90.2 ± 4.4	c	a
	Mixed culture	Control	92.3 ± 7.0		a
		5-deoxystrigol	96.5 ± 4.3		a
		GR24	95.1 ± 9.5		a

Letters in the rightmost columns indicate significant differences in mean germination rate (Tukey *p* < 0.05). For the Across Strain comparisons only monoculture plates are analyzed. For Within Strain comparisons letters are only compared within each focal strain.

### 3.2 Effect of intraspecific interactions on germination in the presence of strigolactones

We then investigated whether inter-strain interactions affected the spore germination in the presence of strigolactones (Figure 3, Table 1). For C2 spores, germination rate was not affected by either strigolactone treatment, or the presence of A5 spores or their interaction (all factors *p* > 0.05, Figure 3B). For A5 spores there was a significant effect of germinating in mixed vs. monoculture (partner effect, 𝜒^2^ = 22.96, 𝑑𝑓 = 1, *p* < 0.0001) but the lack of interaction shows that this was comparable across all strigolactone treatments (partner × treatment, 𝜒^2^ = 3.255, 𝑑𝑓 = 2, *p* = 0.1964). This is demonstrated by observed reductions in germination probability in mixed culture relative to monoculture (control: 37 to 28%; 5-deoxystrigol: 71 to 55% GR24: 80 to 56%, Figure 3A).

### 3.3 Effect of strigolactones and mixture on total hyphal length

**Figure 4:**
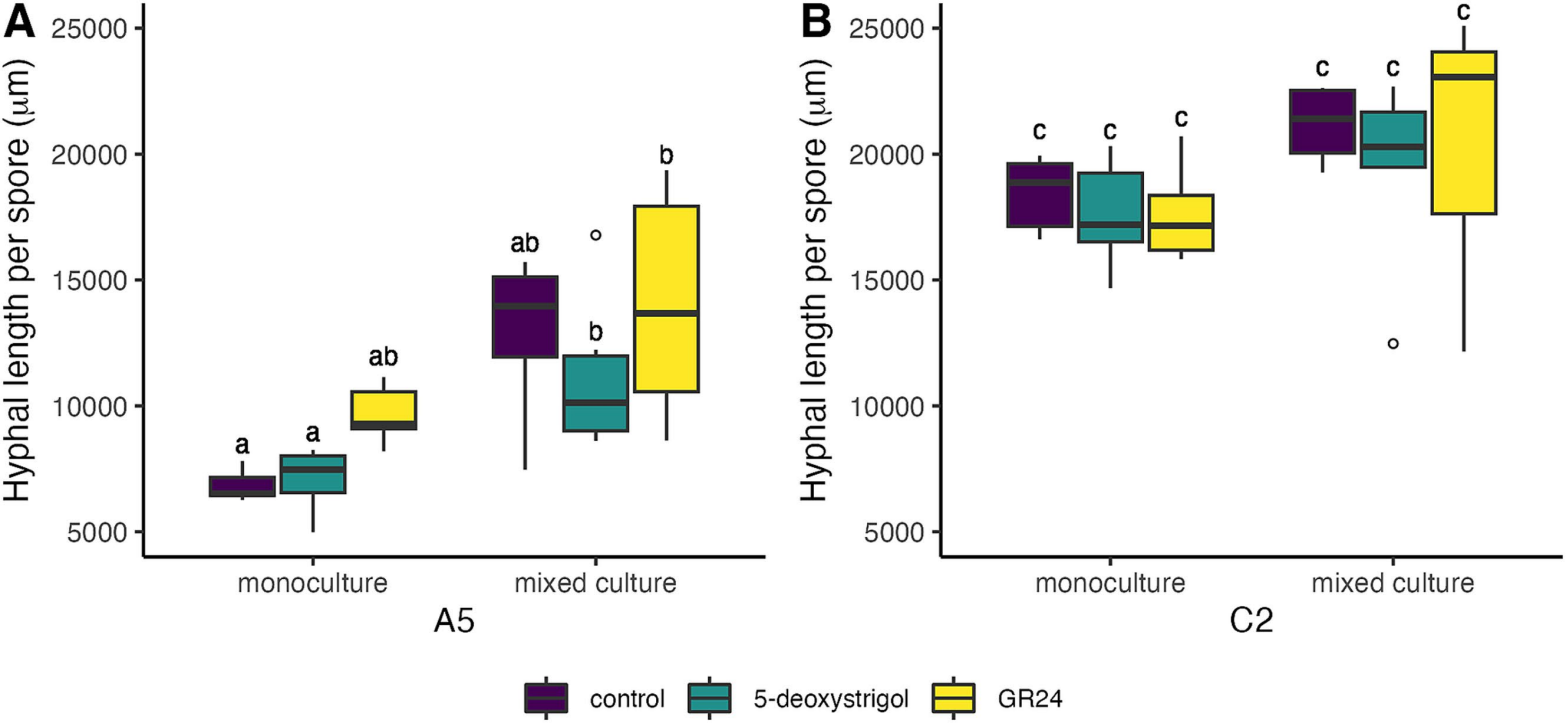
Hyphal length per spore of *R. irregularis* A5 (A) and C2 (B) 7 days after germination in response to 5-deoxystrigol (green), GR24 (yellow), or in the control (purple), in mixed or monoculture conditions. Different letters indicate pairwise statistical differences (*p* < 0.05, Tukey post-hoc test, *n* = 6 for all groups) The boxplots (*n* = 6 for each group) show the first and third quartile (boxplot edges), median (dark line), and data range (whiskers). Open circles show data outliers.

We further examined the effect of strigolactones on pre-symbiotic growth, specifically the effect on total hyphal length (Figure 4 and Table 2; Supplementary Table 1). We found that strain identity (*F* = 204.3, *df* = 1, *p* < 0.0001) and mixture (*F* = 27.5, *df* = 1, *p* < 0.0001), but not treatment (*F* = 1.7, *df* = 2, *p* = 0.2) had a significant effect on the total hyphal length per germinated spore at 7 days post germination. There was no significant interaction among any of these factors (all interaction terms *p* > 0.05). Averaged over treatment and mixture, germinating spores of C2 produced approximately 86% longer hyphae compared to A5 spores. When focusing on each strain individually, germinating A5 spores showed a significant response in hyphal length to mixture and a weak but significant response to treatment, with no significant interaction (treatment: *F* = 3.3, *p* = 0.05; mixture: *F* = 29.4, *p* < 0.0001; treatment × mixture: *F* = 0.4, *p* = 0.6). When averaged over all three treatments, hyphal length of germinating spores in plates of mixed culture (12,721 μm [11,424–14,017]) were higher than in monoculture (7,856 μm [6,560–9,153]). Note that this is not reflected in the specific pairwise comparisons, presumably due to the reduced power of these tests (Figure 4A). For C2 spores, there was only a significant effect of mixture (treatment: *F* = 0.5, *p* = 0.6; mixture: *F* = 6.5, *p* = 0.02; treatment × mixture: *F* = 0.1, *p* = 0.9). When averaged over all three treatments, hyphal length of germinating C2 spores in plates of mixed culture (20,437 μm [18,991–21,883]) were higher than in monoculture (17,878 μm [16,432–19,324]). As above, this was not reflected in the pairwise comparisons (Figure 4B).

**Table 2:** Hyphal length and branching (per spore per plate +/− standard deviation) 7 days post-germination.

Strain	Mixture	Treatment	Hyphal length per spore (μm)	Branches per spore
A5	Monoculture	Control	6,815 ± 620 a	1.39 ± 0.196 a
		5-deoxystrigol	7,102 ± 1,254 a	1.68 ± 0.119 ab
		GR24	9,652 ± 1,155 ab	2.02 ± 0.167 ab
	Mixed culture	Control	12,980 ± 3,125 ab	2.04 ± 0.497 ab
		5-deoxystrigol	11,147 ± 3,114 b	2.34 ± 0.302 bc
		GR24	14,035 ± 4,556 b	2.73 ± 0.666 c
C2	Monoculture	Control	18,454 ± 1,483 c	2.25 ± 0.216 d
		5-deoxystrigol	17,601 ± 2,133 c	2.91 ± 0.488 de
		GR24	17,579 ± 1,852 c	2.86 ± 0.47 de
	Mixed culture	Control	21,200 ± 1,530 c	2.19 ± 0.209 d
		5-deoxystrigol	19,512 ± 3,676 c	3.47 ± 0.801 e
		GR24	20,598 ± 5,300 c	3.43 ± 0.578 e

Letters in indicate significant differences (Tukey *post-hoc*, *P* < 0.05). Comparisons were carried out within strains only.

### 3.4 Effect of strigolactones and mixture on branching

**Figure 5:**
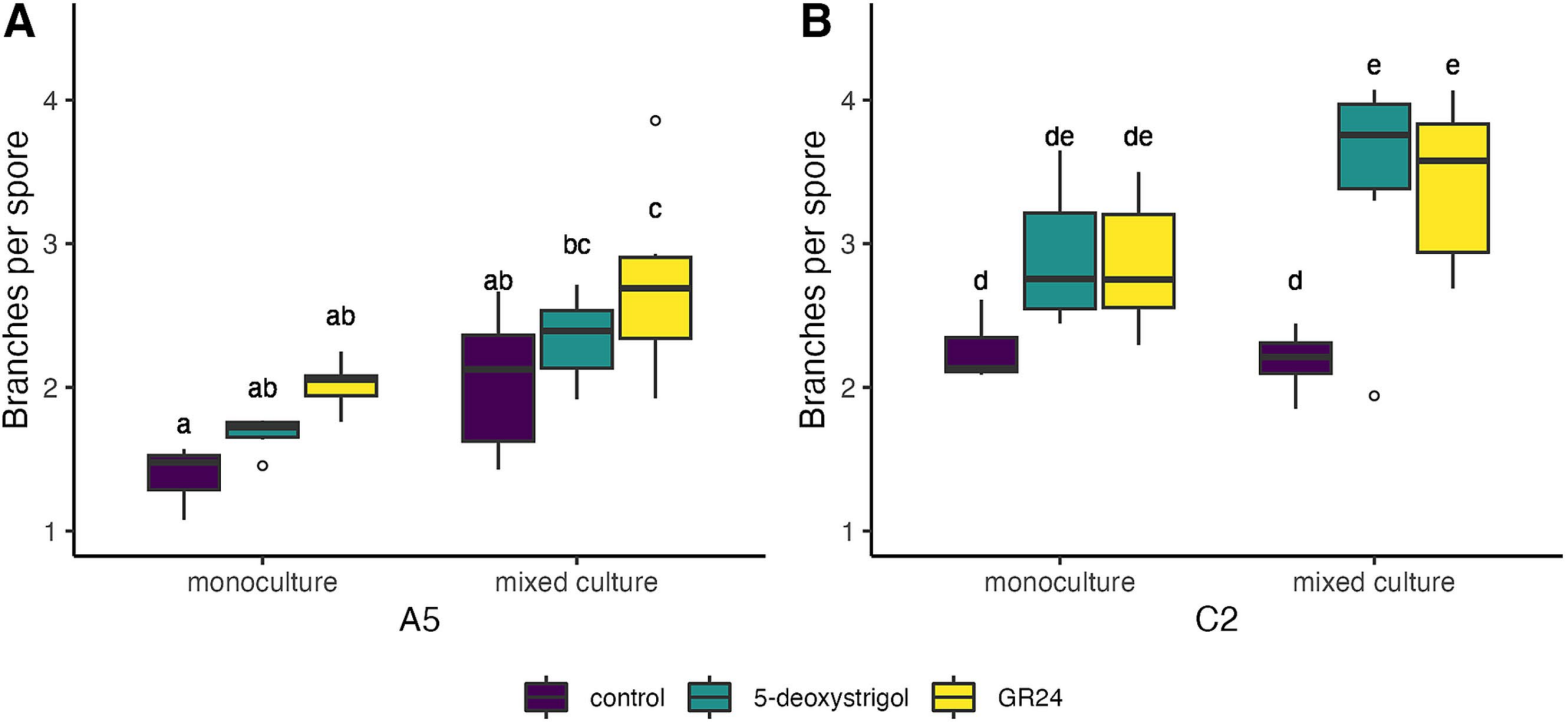
Branches per spore of *R. irregularis* A5 (A) and C2 (B) 7 days after germination in response to 5-deoxystrigol (green), GR24 (yellow), or in the control (purple), in mixed or monoculture conditions. Different letters indicate pairwise statistical differences (*p* < 0.05, Tukey post-hoc test, *n* = 6 for all groups). The boxplots (*n* = 6 for each group) show the first and third quartile (boxplot edges), median (dark line), and data range (whiskers). Open circles show data outliers.

We lastly explored the effect of strigolactones on pre-symbiotic architecture, namely the branches produced per spore (Figure 5 and Table 2). We observed that strain identity (*F* = 60.8, *df* = 1, *p* < 0.0001), treatment (*F* = 20.7, *df* = 2, *p* < 0.0001), and mixture (*F* = 60.8, *df* = 1, *p* < 0.0001), all had a significant effect on branches per germinated spore at 7 days post germination. There was no significant interaction among any of these factors (all interaction terms *p* > 0.05). Averaged over treatment and mixture, germinating spores of C2 produced approximately 40% more branches compared to A5 spores. When focusing on each strain individually, germinating A5 spores showed a significant response in hyphal branching to both treatment and mixture, with no significant interaction (treatment: *F* = 9.0, *p* < 0.0001; mixture: *F* = 28.4, *p* < 0.0001; treatment × mixture: *F* = 0.02, *p* = 0.97). This was mostly driven by the higher number of branches per spore in cultures treated with GR24 (2.73 [2.41–3.04]) compared to spores in control conditions (2.04 [1.72–2.36], Figure 5A) when grown in mixed culture. A similar pattern was observed in monoculture plates, however this was only marginally significant (*p* = 0.07, Figure 5A). For C2 spores, there was also a significant effect of treatment and mixture with no interaction (treatment: *F* = 14.2, *p* < 0.0001; mixture: *F* = 4.5, *p* = 0.04; treatment × mixture: *F* = 1.5, *p* = 0.2). This is reflected in the higher number of branches of spores treated with 5-deoxystrigol (3.47 [3.05–3.89]) or GR24 (3.43 [3.01–3.85]) compared to controls (2.19 [1.77–2.61]) in mixed culture (Figure 5B). The spores in monoculture showed a similar pattern of response to medium treatment but these differences were not identified as significant in the pairwise comparisons (Figure 5B).

## 4 Discussion

AMF mycelia are coenocytic (i.e., aseptate) networks in which thousands of nuclei move freely. In homokaryotic strains such as *R. irregularis* C2, all nuclei are genetically identical. In dikaryotic strains such as *R. irregularis* A5, nuclei of two genetically distinct types exist. These two nucleotypes vary in ratios without ever fully eliminating one or the other (Kokkoris et al., 2020; 2021; Cornell et al., 2022). Although they belong to the same species, homo-and dikaryotic strains exhibit strong differences in genetic variation, as well as life history traits regarding germination, colonization, and foraging behavior (Hart et al., 2001; Serghi et al., 2021; Chen et al., 2018; Yildirir et al., 2022; Sperschneider et al., 2023).

In this study, we tested the effects of intraspecific interactions of *R. irregularis* strains A5 and C2 in the presence of the strigolactone 5-deoxystrigol and the synthetic strigolactone analogue GR24 on a selection of life history traits. We focused on spore germination and the pre-symbiotic life history traits total hyphal length and branches per germinated spore. Our results revealed that strain identity, strigolactone treatment, and presence of another strain influenced the magnitude of germination rate of *R. irregularis* spores. Furthermore, we also found that strain identity and the presence of another strain affected hyphal length and branching. Overall, strigolactones were observed to affect life history traits differently for the two strains.

### 4.1 Strain identity impacts germination rate and determines the magnitude of germination response in the presence of strigolactones

In line with previous findings (Serghi et al., 2021), we confirmed that homokaryon C2 germinated faster and to a greater extent than the dikaryon A5 (Figure 2). Our results further showed that both strigolactones significantly increased the germination of the dikaryotic strain A5, whereas they did not affect the already high germination of homokaryotic strain C2 (Figures 2, 3) highlighting the possibility that some strains might remain unaffected by specific strigolactones during germination. But because of the constant high germination rate of C2 regardless of the strigolactone treatment, the effect of strigolactones on C2 spore germination cannot be properly assessed. Interestingly, the application of strigolactones did not reduce the time between exposure and germination. Serghi et al. (2021) hypothesized that the difference between germination time of homokaryons and dikaryons may result from initial nuclei coordination difficulties; since dikaryons contain two nucleotypes, they may first have to overcome inter-nucleus genetic interactions to initiate germination. The same authors hypothesized as an alternative that maintenance of two nucleotypes with unique protein expression may lead to increased metabolic costs for the spore. This increased metabolic cost may in turn delay the germination of dikaryotic strains compared to homokaryotic strains.

In addition to inter-nuclei genetic conflict, general intraspecific genetic variation of strains may play an important role in explaining differences between homokaryon and dikaryon responses. It has recently been shown that the intraspecific genetic variation of *R. irregularis* strains is much larger than initially believed. Even the two nucleotypes within a single dikaryotic strain are highly diverse with multiple unique genes (Chen et al., 2018; Yildirir et al., 2022; Sperschneider et al., 2023). Moreover, *R. irregularis* harbors highly heterogeneous, non-tandemly repeated rDNAs that are all actively transcribed and may thus modulate translation in response to different biotic and abiotic factors (Maeda et al., 2018). It is therefore feasible that strain-specific variation in sensitivity to chemical signals such as strigolactones has a basis in this (epi-)genetic variation.

It remains to be investigated whether dikaryon germination is delayed and decreased compared to the homokaryon due to nucleotype coordination. The strigolactones might provide a signal for the spore to, for example, induce synchronized favorable gene expression (Hua et al., 2024; Thula et al., 2023; Tsuzuki et al., 2016), resulting in an overall increased number of successfully germinating spores. However, since the germination delay remained unchanged, we could assume that strigolactones do not reduce all potentially existing inter-nuclei genetic conflict in dikaryons *per se*.

In summary, we found that the strain identity impacts the germination rate and determines the magnitude of germination response in the presence of strigolactones. The homokaryon C2 germinated faster but the effect of strigolactones could not be properly assessed. Application of strigolactones increased the germination rate of the dikaryon A5 but did not reduce the time to germination. Follow-up experiments are required to elucidate if the observed differences are a result of inter-nucleus coordination difficulties, intraspecific genetic variation, or other yet unknown processes. These experiments should include multiple homokaryotic and dikaryotic strains and should investigate gene and protein expression levels across the distinct groups in the presence and absence of strigolactones.

### 4.2 Presence of C2 spores reduces the A5 spore germination

We found that germinating *R. irregularis* spores from two distinct strains reduced the rate of spore germination for strain A5. This effect was observed for all strigolactone treatments and control conditions, and led to reductions ranging from approximately 10 to 25 percentage points indicating that intraspecific competition seems the primary reason for this response. The exact mechanism behind this effect remains unclear. A possible explanation could be that C2 produces secondary metabolites as inhibitory compounds that hinder other spores’ germination. The reduced germination rates of neighbouring strains would then result in an advantage for individual C2 spores to successfully colonize a root and enter the symbiotic partnership.

Microorganisms widely use volatile and non-volatile organic compounds as means to inhibit or kill competitors (Garrett and Robinson, 1969; Granato et al., 2019). In the fungi kingdom, compounds such as peptides, aromatic alcohols, lipids, and volatile organic compounds are involved in for example quorum sensing (Cottier and Mühlschlegel, 2012; Mehmood et al., 2019). Furthermore, aromatic alcohols such as tyrosol and farnesol produced by yeast *Candida albicans* can have self-regulatory functions: tyrosol promotes germ tube formation in the yeast (Chen et al., 2004), whereas farnesol inhibits its filamentous growth (Hornby et al., 2001; Oh et al., 2001). These compounds also affect other fungal species in various ways that imply evidence for antagonistic mechanisms in fungi: farnesol inhibits germination and growth of the filamentous fungi *Fusarium graminearum* (Semighini et al., 2008) and *Penicillium expansum* (Liu et al., 2009) and can even induce apoptosis in *Aspergillus niger* (Semighini et al., 2006). Moreover, species-specific auto-signaling properties of aromatic alcohols have been reported in yeast: tryptophol and phenylethanol promote pseudo hyphal growth in *Saccharomyces cerevisiae*, but they do not stimulate filamentous growth in *C. albicans* (Chen and Fink, 2006). Given the prevalent occurrence of self-regulatory and antagonistic compounds in fungi, investigating the (strain-specific) secondary metabolites exuded by germinating AMF spores may provide insight in the mechanism at play.

Despite the importance of secondary metabolites in interspecific interactions, little is known about secondary metabolites from AMF and their involvement in *intra*specific interactions. Germinating AMF spores have been reported to produce phytohormones, such as cytokinins, auxins and ethylene, to interact with the plant host and/or for their own developmental regulation (Pons et al., 2020). For instance, in low concentrations, AMF-produced ethylene promotes spore germination while it inhibits it when exceeding a certain threshold (Ishii et al., 1996). Other diffusible signaling molecules from germinating AMF spores have been reported to elicit molecular responses in plants, such as cytosolic calcium signaling (Navazio et al., 2007), starch accumulation (Gutjahr et al., 2009), and developmental changes in root architecture (Mukherjee and Ané, 2010). While *inter*specific interactions of AMF have been shown to negatively affect plant growth (Violi et al., 2007; Malicka et al., 2021) and host nutrient acquisition (Horsch et al., 2023), data showing *intra*specific interaction effects of AMF are lacking. Fungal metabolites produce during germination could have important implications for the priority effects of AMF during root colonization by providing advantages or disadvantages for different AMF species and/or strains.

In summary, we found that the presence of C2 spores reduce the germination rates of A5 spores. Follow-up studies are required to investigate the specific compounds and mechanisms involved. These studies should furthermore include a broader selection of *R. irregularis* strains to investigate the role of different nucleotypes of homokaryons and dikaryons. A particularly promising approach could be to isolate and characterize what volatile or non-volatile compounds are exuded by the germinating spores of the strains, using metabolomics. Differential compounds could be isolated and tested in different concentrations to determine their effect on spore germination (Mukherjee and Ané, 2010).

### 4.3 Strigolactones and cultivation in mixed cultures increase branching

We demonstrated that both strains produce more branches in response to strigolactone exposure when cultivated in mixture (Figure 5). Increased branching allows the fungus to explore the environment in multiple directions simultaneously. Consequently, exploring multiple directions may increase the chances of intercepting and successfully colonizing a root to establish the symbiosis. The increased branching per spore in response to strigolactones may therefore be directly linked to increasing the fitness of the spore.

In both strains, branching is increased in response to strigolactone treatment when the spores germinate in a mixed culture. Interestingly, although a trend of increased branching per spore can be observed in both mixture conditions (monoculture and mixed culture), this is only significant in mixed cultures. This indicates that it is not only plant-derived signals such as strigolactones that affect pre-symbiotic growth of AMF, but that exudates of surrounding spores (Mukherjee and Ané, 2010) might also play a role in the pre-symbiotic growth response. It remains to be investigated whether these potential exudates of germinating spores are volatile or non-volatile compounds, and how they act on the pre-symbiotic growth response of spores of other strains or species.

While the homokaryon C2 similarly responded to both strigolactones equally in mixed cultures, the dikaryon A5 significantly responded only to GR24. This could be linked to the aforementioned large genome variability between *R. irregularis* strains and the consequent unique selection of genes (Chen et al., 2018; Yildirir et al., 2022; Sperschneider et al., 2023). Similarly, the aforementioned highly heterogeneous rDNAs in *R. irregularis* (Maeda et al., 2018) could result in a dynamically modulated translation machinery. Our current hypothesis is that A5 and C2 differ in their ability to perceive strigolactones by for example expressing different receptors, or that they vary in the signaling pathway downstream of the strigolactone-perception.

It is worth noting that the stability of the strigolactones in aqueous conditions could have partially affected these results for A5. The synthetic GR24 has a longer half-life of 10 days in water compared to the half-life of 1.5 days for 5-deoxystrigol in water (Akiyama et al., 2010). A5 may be responding less in hyphal branching due to a slight mismatch of the strain’s germination time of more than four days (Figure 2) and the half-life of the strigolactone. The initial concentration of 5-deoxystrigol was sufficient to trigger an increased A5 germination rate (Figure 3), but the availability of non-degraded 5-deoxystrigol in the medium decreased over time. At the emergence of A5 germ tubes after four days, the concentration of non-degraded 5-deoxystrigol may have been insufficient to elicit a response. C2’s increased production of hyphae in response to both strigolactones may be linked to its quicker germination compared to A5. Future work could explore application of strigolactones at different time points to overcome the temporal instability of these chemicals.

In summary, it is unclear which factors drive the differences regarding the increase of branching of hyphae of A5 and C2 in response to strigolactones. It remains to be investigated whether the observations can be attributed to varying strigolactone stability, competing spores’ exudates, or genetic differences of the strains. A genetic difference resulting in for example strigolactone receptor variation or variation in the downstream signaling pathway could be investigated in the future by including multiple homo-and dikaryotic strains.

### 4.4 Cultivation in mixed culture increases hyphal length in A5

Overall, we show that hyphal length of germinating spores remained largely unaffected by the two applied strigolactones in our study. However, A5 spores that germinated in the presence of C2 produced significantly more hyphae. This finding is interesting when evaluated in combination with our result showing that C2 reducing the germination potential of A5 in the strigolactone treatments. Although A5 spores germinate more when treated with strigolactones, they do not significantly increase their hyphal length. When spores of C2 are present, A5 spore germination rate remains below its full potential. However, the (fewer) A5 spores that germinate, produce longer hyphae.

Increasing branching and total hyphal length, and thus biomass, in response to potential competition might be an A5 strain trade-off between its low germination percentage / slower germination start and extent of exploration. An alternative hypothesis would be that the induced asymmetric changes in A5 hyphal growth might be related to pre-fusion hyphal incompatibility with the neighboring strain C2 as an attempt to dominate over a potentially competitive strain (Kennedy, 2010). C2 shows the highest germination rate and hyphal length regardless of the presence or absence of strigolactones. Therefore, in this oversimplified system, C2 may appear to be the winner in the race toward successful root colonization, despite A5’s stronger responsiveness to plant signals. However, when the different germination strategies of A5 and C2 are placed in an ecological context, both have their advantages and disadvantages. Despite priority effects, more than one AMF can colonize a root or host (Werner and Toby Kiers, 2015), so there is a limited risk attached to the strategy of A5. Increasing its germination at the presence of strigolactones may give other, less plant signal sensitive, strains such as C2 a head start when it comes to root colonization, but it increases survival chances of the focal strain. Most importantly, AMF germlings can also survive by directly connecting to a pre-existing network of a kin strain (Sbrana et al., 2011; Purin and Morton, 2013). Consequently, by responding to strigolactones, the strain also increases its chances to intercept and connect to a pre-existing hyphal network and therefore expand faster and further compared to hyphal networks that establish from a single spore. Therefore, a higher responsiveness to strigolactones could potentially increase the chances of survival regardless of other strains nearby. A direct benefit over C2 may be that A5 increasingly germinates when suitable hosts are around, and therefore persists in the soil for a longer period. C2, which is not responsive to strigolactones regarding germination, might germinate in unfavorable conditions (i.e., no host available) and therefore die out. However, the germination strategy of C2 may prove beneficial in densely plant-covered environments in which roots are always present and available to the fungus.

The research on intraspecific competition of AMF in the context of strigolactones is still in its infancy and simple, controllable environments are required to start filling the knowledge gaps. This study uses an oversimplified system to investigate complex intra-specific dynamics of natural environments. Once more understanding of the intricate relations and interactions between strains is acquired, the next step will be to gradually increase system complexity. One way of adding components to the system toward a more “natural” environment may be the application of “Transparent Soil,” a moisture-absorbent polyacrylate polymer that allows for non-sterile cultivation of AMF (Paré et al., 2022). This allows researchers to bypass the surface sterility requirement of conventional *in vitro* systems while retaining the transparency of the medium. This novel culturing technique will allow to closely monitor germination and pre-symbiotic responses of AMF species that are normally excluded from *in vitro* studies.

## 5 Conclusion

Our findings provide evidence that there are AMF strain-specific reactions to strigolactones which are in some circumstances mediated by interactions between strains. In particular, we found that the strigolactone-related germination responses differed strongly between strains. This raises the possibility that when using single model strains when assessing effects of strigolactones crucial dynamics may be overlooked. Together with findings of previous studies, our results emphasize that several different phenotypes need to be analyzed when studying AMF responses to strigolactones. As plants produce genus-and species-specific strigolactone blends, their degree of physiological response by AMF could be strain-specific. Our data suggest that AMF strain-specific responses to strigolactones could have the potential to shift the colonization advantage determined by priority effects from one AMF species to another. This may have direct implications for the establishment of the AMF symbiotic community. However, the degree to which strain interactions and reactions to specific strigolactones play a role in shaping the community remains unclear.

## Data Availability

The raw data supporting the conclusions of this article will be made available by the authors, without undue reservation.
