# Supplementary material for: The potential of strigolactones to shift competitive dynamics among two Rhizophagus irregularis strains
Source: Front Microbiol. 2024 Oct 18;15:1470469. doi: 10.3389/fmicb.2024.1470469 (PMC11524933; doi:10.3389/fmicb.2024.1470469)
Supplement: Supplementary file 1 [file Table_1.DOCX]

Supplementary Material

**Supplementary Figure 1.** Pre-symbiotic hyphae of *Rhizophagus irregularis* strains A5 (left column) and C2 (right column) in the control (top row), 5-deoxystrigol (middle row), and GR24 treatment (bottom row) 14 days after inoculation. Contrast has been adjusted for better visibility of the hyphae.
